# Supplementary material for: Identification of the Genes Encoding B3 Domain-Containing Proteins Related to Vernalization of Beta vulgaris
Source: Genes (Basel). 2022 Nov 25;13(12):2217. doi: 10.3390/genes13122217 (PMC9778101; doi:10.3390/genes13122217)
Supplement: Supplementary file 1 [file genes-13-02217-s001.zip › Supplemental Figures and Legends.pdf]

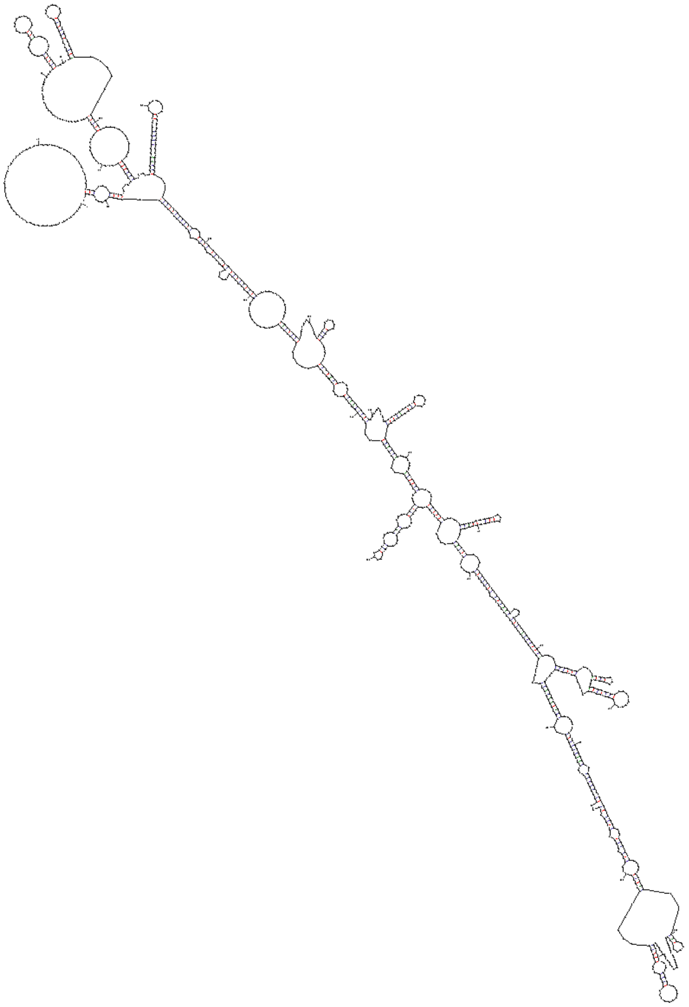

Figure S1. The secondary structure of long noncoding RNA *MSTRG.26204* of *Beta vulgaris*.

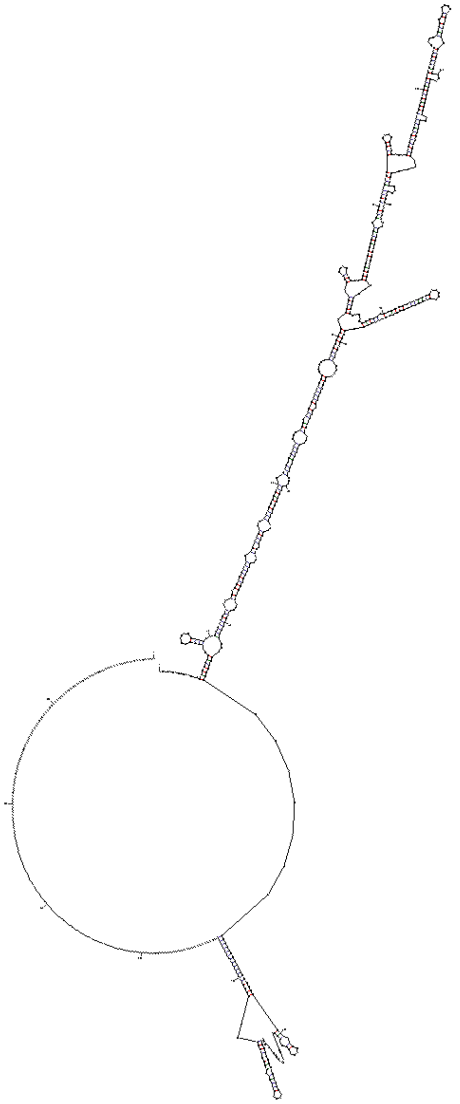

Figure S2. The secondary structure of long noncoding RNA *COOLAIR* of *Arabidopsis thaliana*

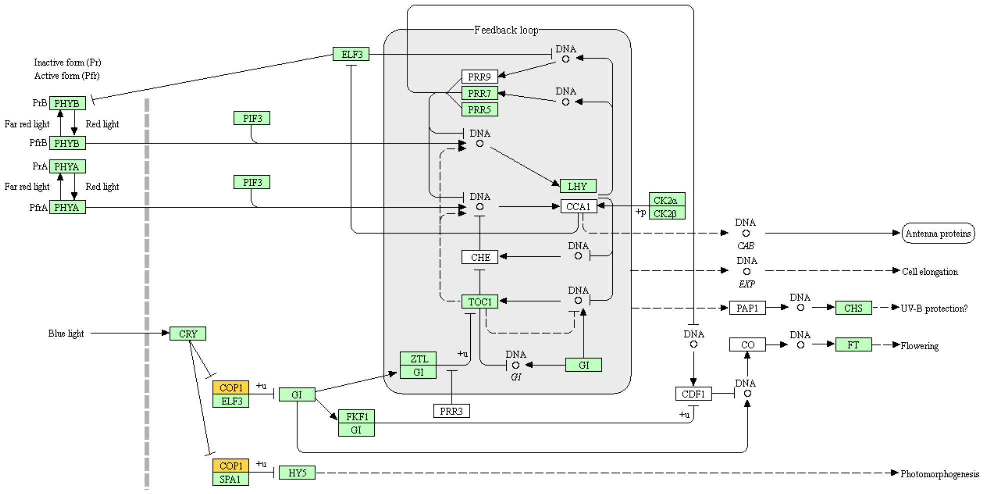

Figure S3. Identified the circadian rhythm pathways of *Beta vulgaris*.

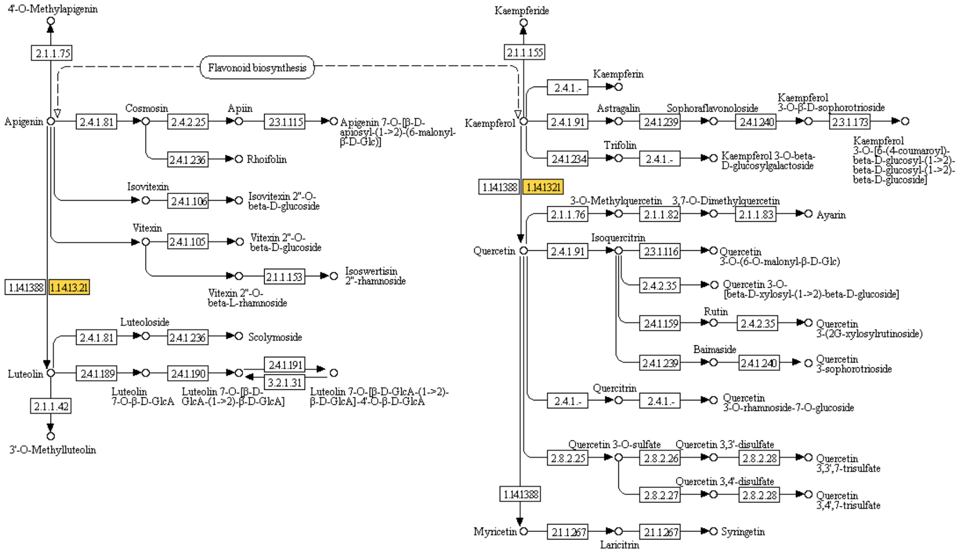

Figure S4. Identified the flavone and flavonol biosynthesis pathways of *Beta vulgaris*

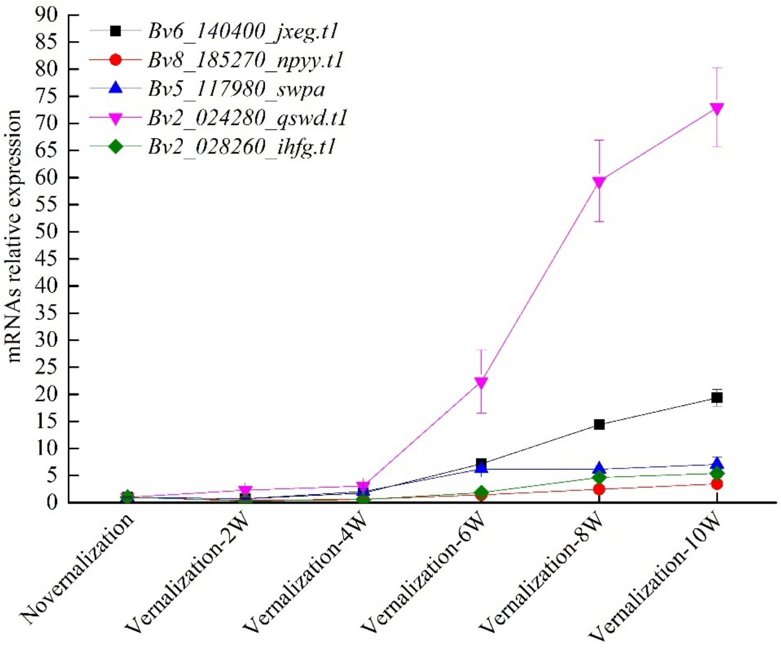

Figure S5. A

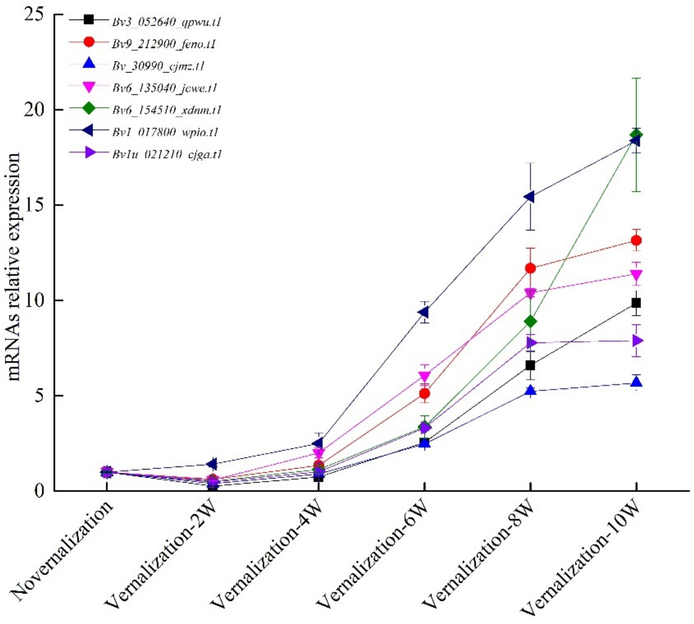

Figure S5. B

Figure S5. Quantitative real-time polymerase chain reaction (qRT PCR) analysis. qRT-PCR results for (A) differentially expressed mRNAs; (B) *Bv3_052640_qpwu.t1*, *Bv9_212900_feno.t1* and *Bv_30990_cjmz.t1* were zinc finger CCCH domain-containing proteins. *Bv6_154510_xdnm.t1* was zinc finger MYND domain-containing protein. *Bv6_135040_jcwe.t1*, *Bv1_017800_wpio.t1* and *Bv1u_021210_cjga.t1* were F-box proteins.

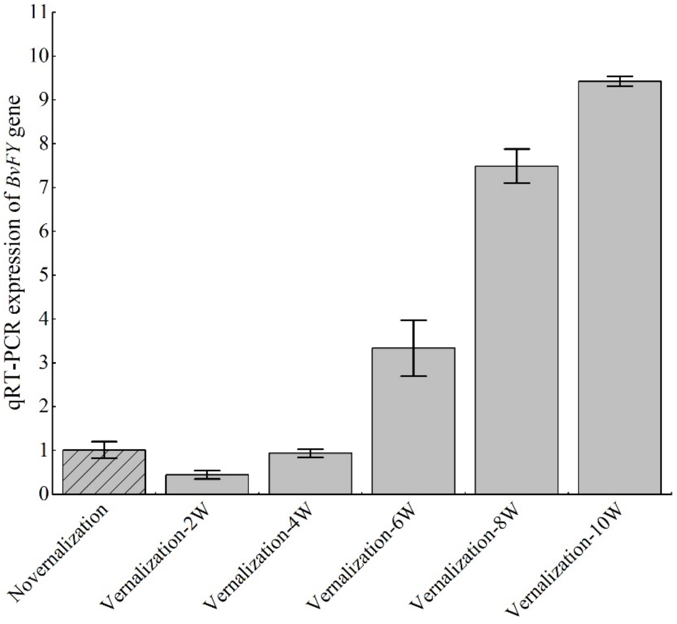

Figure S6. A

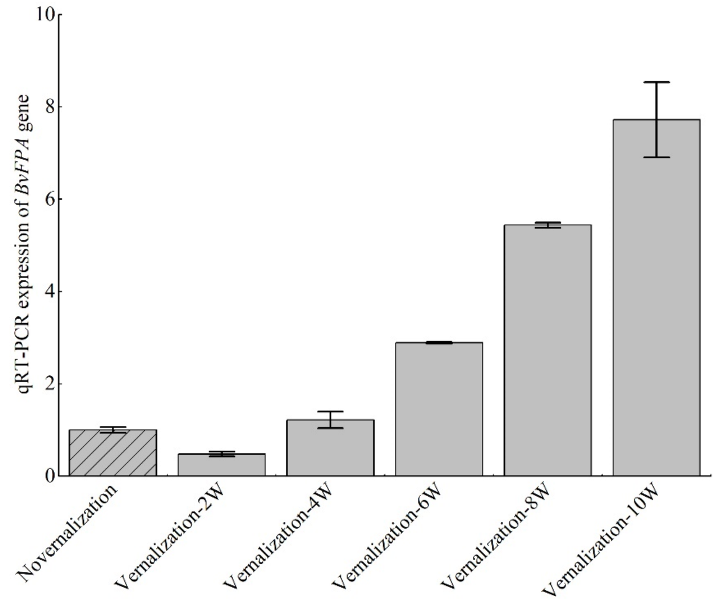

Figure S6. B

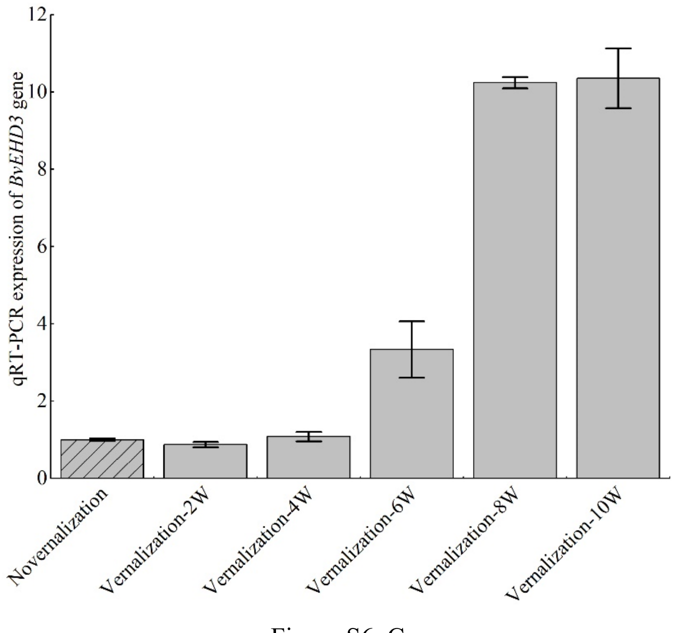

Figure S6. C

Figure S6. Quantitative real-time polymerase chain reaction (qRT-PCR) analysis. qRT-PCR results for (A) Bv2_043610_suxu.t1 was the flowering time control protein FY; (B) Bv3_059000_kgww.t1 was the flowering time control protein FPA; (C) Bv1_011260_hdsi.t1was the PHD finger protein EHD3.
